# Fingerprint sign in Vogt-Koyanagi-Harada disease: a case series

**DOI:** 10.1186/s40942-021-00356-y

**Published:** 2022-01-10

**Authors:** Ruy Felippe Brito Gonçalves Missaka, Mauro Goldbaum, Cleide Guimarães Machado, Emmett T. Cunningham, Fernanda Maria Silveira Souto, Marcelo Mendes Lavezzo, Priscilla Figueiredo Campos da Nóbrega, Viviane Mayumi Sakata, Maria Kiyoko Oyamada, Carlos Eduardo Hirata, Joyce Hisae Yamamoto

**Affiliations:** 1 Department of Ophthalmology, LIM-33, Faculdade de Medicina FMUSP, Universidade de São Paulo, São Paulo, SP 01246-000 Brazil; 2 Department of Ophthalmology, Federal University of Paraná, Curitiba, PR Brazil; 3 Brazilian Vogt-Koyanagi-Harada Disease Study Group, São Paulo, SP Brazil; 4 West Coast Medical Group, San Francisco, CA USA; 5 Department of Ophthalmology, California Pacifical Medical Center, San Francisco, CA USA; 6 Department of Ophthalmology, Stanford University School of Medicine, Stanford, CA USA; 7 The Francis I Proctor Foundation, UCSF School of Medicine, San Francisco, CA USA

**Keywords:** *En face* OCT, Inflammation, Uveitis, Uveomeningoencephalitic syndrome

## Abstract

**Background:**

The tomographic finding, which has been called the "fingerprint sign" in *en face* reconstructions, seems to be the result of a variety of processes that cause distension of the outer plexiform layer (OPL) and the Henle fiber layer (HFL). The aim of this paper is to describe the appearance of concentric rings at the OPL/HFL interface visualized using *en face* reconstructions of cross-sectional optical coherence tomography images of patients with Vogt-Koyanagi-Harada disease.

**Methods:**

Retrospective analysis of images of six eyes of three patients obtained by cross-sectional OCT imaging and *en face* reconstruction at the level of the OPL/HFL interface.

**Results:**

All eyes presented with a *dentate* or *saw-tooth* pattern of the OPL/HFL interface on cross-sectional OCT with corresponding concentric rings on *en face* OCT reconstruction, consistent with the recently published “fingerprint sign”. Initial OPL/HFL interface changes were observed between the first and fourth months after treatment and resolution of VKHD associated serous retinal detachments. These OPL/HFL interface changes have persisted for many years following the resolution of the active inflammation.

**Conclusions:**

Changes in the OPL/HFL interface can be identified following successful treatment of VKHD. These included both a *dentate* or *saw-tooth* pattern on cross-sectional imaging and concentric rings or the “fingerprint sign” on *en face* reconstructions. These changes persisted for many years despite disease quiescence.

## Background

Vogt-Koyanagi-Harada disease (VKHD) is a systemic granulomatous autoimmune disease that targets melanocyte-rich tissues, including the eye, inner ear, meninges, skin, and hair follicles. Ocular involvement in VKHD is characterized by severe bilateral granulomatous panuveitis associated with serous retinal detachments (SRDs) and optic disc hyperemia/edema, with eventual development of a sunset glow fundus [1, 2].

Understanding of VKHD has improved greatly with the use of optical coherence tomography (OCT). In the acute phase of VKHD, OCT findings include thickening of the choroid that results in undulation of the retinal pigment epithelium (RPE)-Bruch’s membrane complex, multifocal SRDs, often with sub-retinal *septae*, discontinuity or disruption of ellipsoid zone and interdigitation zone lines, and the presence of amorphous hyper-reflective material suggestive of fibrin admixed with shed photoreceptor inner and outer segments [3–5]. Agarwal et al. proposed that sub-retinal *septae* in the setting of SRD and VKHD represent a bacillary layer detachments, or a shearing of the photoreceptor layer at the level of inner/outer segments, consistent with previous observations [5, 6]. There have been few studies of the OCT findings in the convalescent phase of VKHD. In a cross-sectional study assessing late-stage VKHD subjects using spectral domain OCT, Zhou et al. described that defects in the ellipsoid and interdigitation zones can have different recovery patterns, impacting the visual prognosis, and are related to the time of treatment initiation [7].

Recently, the “fingerprint sign” was observed and reported at the level of the outer plexiform/Henle fiber layer (OPL/HFL) interface in subjects with vitreoretinal traction using *en face* reconstructions of cross-sectional OCT images [8]. These concentric, fingerprint-like rings or waves are associated with a *dentate* or *saw-tooth* pattern of the OPL/HFL interface on cross-sectional OCT. The authors hypothesized that these changes in the OPL/HFL interface resulted from disruption of the normally well-organized photoreceptor axons and their associated Müller cells caused by irregularly applied tractional or mechanical forces. They recognized, however, that the “fingerprint sign” might not be limited to vitreoretinal tractional disorders and cited the description of the same sign in foveal hypoplasia [9].

In this retrospective case series, we described six eyes of three patients with VKHD in the convalescent phase, each of whom was found to have disruption of the OPL/HFL interface seen as a *dentate* or *saw-tooth* pattern on cross-sectional OCT imaging and as concentric rings or waves (the “fingerprint sign”) on *en face* OCT reconstruction at the level of the OPL/HFL. The pathogenesis of these findings and the implications for other inflammatory chorioretinopathies are discussed.

## Methods

A retrospective analysis of 6 eyes of 3 VKHD subjects in whom a *dentate* or *saw-tooth* pattern of the OPL/HFL interface on cross-sectional OCT was detected was performed. *En-face* OCT reconstructions were reviewed for the presence of the “fingerprint sign”. This study was conducted at the Uveitis and Retina and Vitreous Services, Hospital das Clínicas da Faculdade de Medicina da Universidade de São Paulo, HCFMUSP, São Paulo, Brazil.

Optical coherence tomography and enhanced depth imaging (EDI-) OCT were performed using scanning spectral-domain (SD-) OCT (Spectralis® HRA + OCT, Heidelberg Engineering, Germany). From 2019, OCT angiography (OCT-A; Spectralis® OCT II, Heidelberg Engineering, Germany) was included in the multimodal analysis. Images with SD-OCT volume scans (20° × 20° with 25 horizontal sections, minimum ART 25) and the 6 mm *horizontal line* scan through the fovea were analyzed. *En face* OCT angiography (OCTA) was evaluated to analyze the changes seen in the OPL/HFL interface on B-scan. The images generated by the *en face* OCT were manually positioned between 95 and 119 µm above of the retinal pigment epithelium, with slab thicknesses ranging between 10 and 30 µm. The contrast and brightness of the images were manually adjusted to improve the visualization of the OPL/HFL interface changes. The study followed the statements of the Declaration of Helsinki and was approved by the local Institutional Review Board (CAAE 29270620.7.0000.0068).

## Results

Six eyes of three subjects with VKHD were identified by a chart review. All subjects were female and the ages at presentation were 26, 39, and 42 years. Initial vision in the six affected eyes ranged from counting fingers to hand motion. Median duration of follow-up was 53 months (range: 43–78 months). In the acute phase, all subjects had prodromal symptoms of headache and/or tinnitus, and signs of aseptic lymphomonocytic meningitis on cerebrospinal fluid analysis. All subjects presented with panuveitis, SRD, and optic disc hyperemia/edema. The time from the onset of symptoms to treatment in the three subjects was 14, 23 and 27 days. All subjects were initially treated with 3-day intravenous methylprednisolone (1 g/day) followed by oral prednisone (1 mg/kg/day) with slow tapering; long-term azathioprine was administered in 2 subjects (cases 1 and 2) and mycophenolate mofetil in 1 subject (case 1). None of the cases had comorbidities. One subject (case 1) had recurrent anterior uveitis; cases 1 and 2 had peripapillary neovascular membrane. Table 1 describes the clinical and epidemiological characteristics of this series of cases.

**Table 1:** Summary of the patient baseline characteristics and clinical features

Case	Eye	Age, years/Gender	Comorbidities	Time treatment, days^1^	Treatment	VA baseline	VA last follow-up	Acute phase^2^	Non-acute phase^3^	Follow-up, months
1	OD	39/F	None	27	Initial high-dose CE and slow tapering AZA at M1 MMF at M24	CF	20/20	Optic disc hyperemia, macular SD	Anterior uveitis recurrence at M24 SGF	43
	OS					HM	20/20	Optic disc hyperemia, macular SD	Anterior uveitis recurrence at M24 CNV at M24 SGF	
2	OD	26/F	None	14	Initial high-dose CE and slow tapering AZA at M1	HM	20/20	Optic disc hyperemia, macular SD	CNV at M12 SGF	53
	OS					CF	20/20	Optic disc hyperemia, macular SD	CNV at M12 SGF	
3	OD	42/F	None	23	Initial high-dose CE and slow tapering	CF	20/20	Optic disc hyperemia, macular SD		78
	OS					CF	20/20	Optic disc hyperemia, macular SD		

^1^Interval between symptoms onset and treatment start

^2^≤ 6 months from disease onset

^3^> 6 months from disease onset

AZA: azathioprine; CE: corticosteroid; CF: counting fingers; CNV: choroidal neovascular membrane; F: female; HM: hand motion; M: month; MMF: mycophenolate mofetil; SD: serous detachment; SGF: sunset glow fundus

The *dentate* pattern on cross-sectional OCT imaging at the OPL/HFL interface was noted at the first month in the case 3, at the third month in the case 2 and at the 6th month in the case 1. This finding was observed throughout the entire follow-up. *En face* OCT examinations were added to the protocol in 2019 and these analyses demonstrated the appearance of the concentric rings or waves at the level of OPL/HFL interface in all three subjects. Both findings spared the center of the foveola and were limited to the perifoveal region consistent with the known location of the OPL/HFL interface. None of these eyes presented signs of posterior vitreous detachment or vitreomacular traction on OCT.

### Case 1

**Fig. 1:**
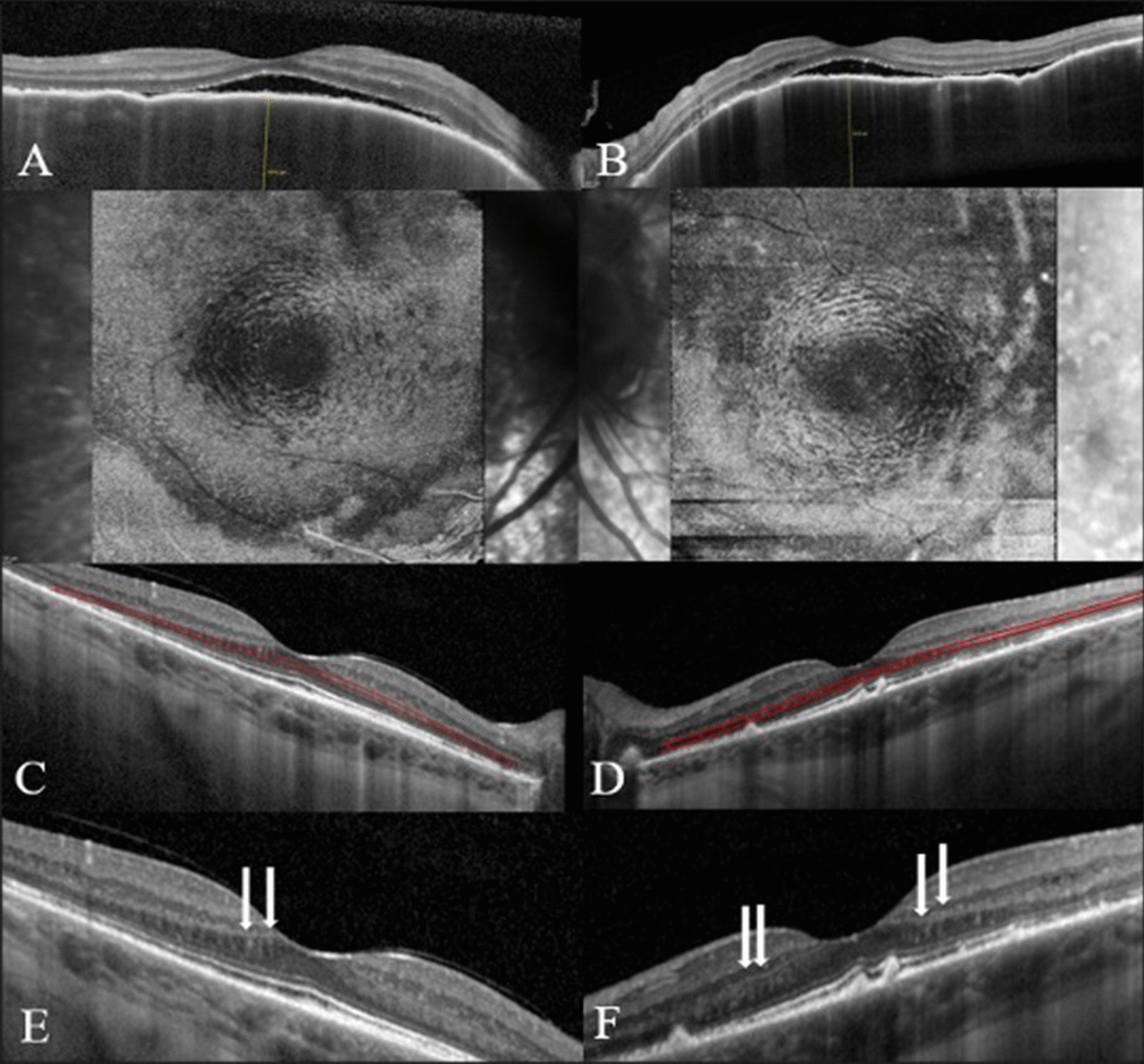
(Case 1): B-scan through the fovea in the acute phase VKH of the right (**A**) and the left (**B**) eye*s.* In the non-acute phase, *en face OCT* shows the "fingerprint sign", and the representative corresponding B-scan at the level of OPL/HFL of the right (**C**) and the left (**D**) eye*s.* The same B-scan through the fovea reveals a *dentate* or *saw-tooth* appearance of the right (**E**) and the left (**F**) eyes (white arrows)

A 39-year-old woman presented with blurred vision OU accompanied by headache and tinnitus for 14 days. Baseline visual acuity was counting fingers OD and hand motion OS. The interval between the onset of symptoms and the start of treatment was 27 days. After treatment with high-dose corticosteroids, visual acuity improved to 20/20 OU by the third month. Azathioprine was introduced in the first month and was used for 2 years, when it was replaced by mycophenolate mofetil due to anterior uveitis (OU), worsening of electroretinographic parameters (OU) and development of peripapillary choroidal neovascular membrane (CNV) in OS. CNV was treated with three-monthly intravitreal bevacizumab injection. The *dentate* appearance at the OPL/HFL interface was first seen in both eyes in the sixth month. The first *en face* OCT reconstruction was performed in the second year of follow-up, demonstrating the concentric rings or waves consistent with the “fingerprint sign” (Fig. 1).

### Case 2

**Fig. 2:**
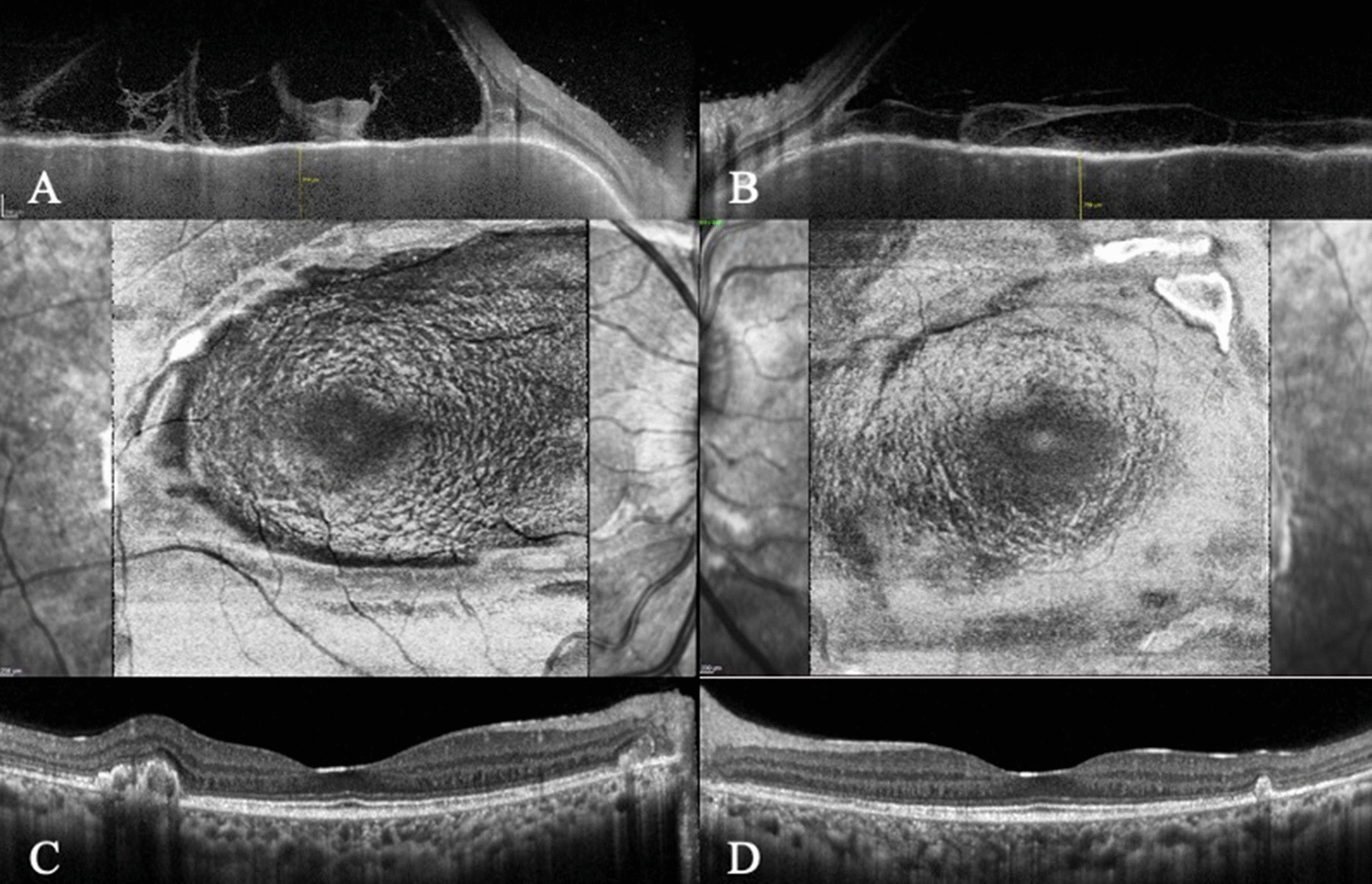
(Case 2): B-scan through the fovea in the acute phase VKH of the right (**A**) and the left (**B**) eye*s.* In the non-acute phase, *en face* OCT shows the "fingerprint sign" and corresponding B-scan at the level of OPL/HFL of the right (**C**) and the left (**D**) eye*s* reveals a *dentate* or *saw-tooth* appearance

A 26-year-old woman presented blurred vision OU accompanied by headache for 10 days. At the time, her visual acuity was hand motion OD and counting fingers OS. There were 14 days between the onset of symptoms and the start of treatment. After treatment with high-dose corticosteroids, visual acuity improved to 20/20 OU by the fifth month. Azathioprine was introduced in the first month. She developed peripapillary CNV OU in the first year of follow-up, treated with four-monthly intravitreal bevacizumab injection OD and three-monthly OS. She subsequently developed subretinal fibrosis in each eye. Currently, she has 20/20 visual acuity OU. The *dentate* appearance OU at the OPL/HFL interface was first seen in the third month of follow-up. In the second year of disease quiescence the first *en face* OCT performed showed the concentric rings or waves characteristic of the “fingerprint sign” (Fig. 2).

### Case 3

**Fig. 3:**
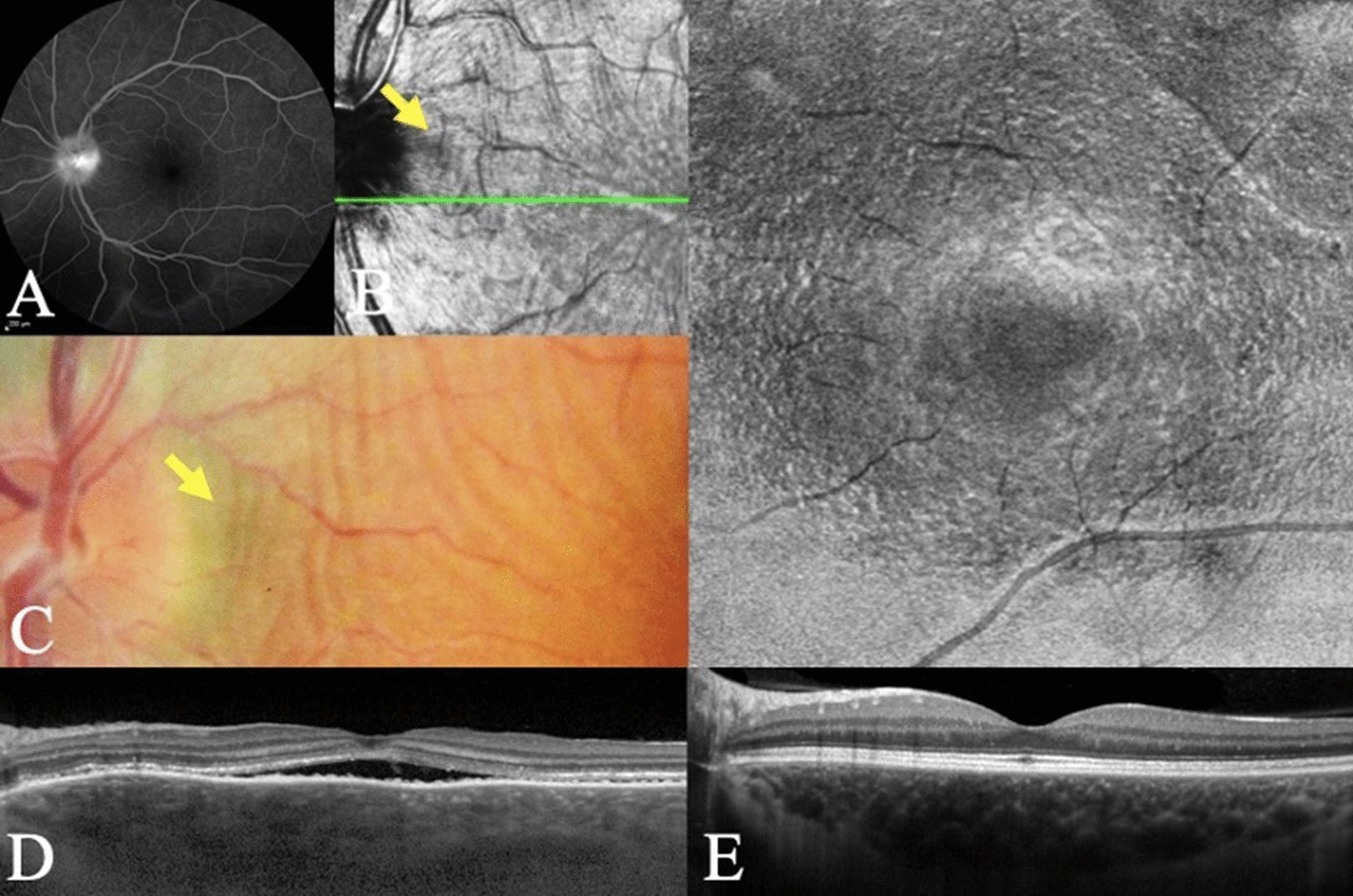
(Case 3): In the acute phase VKH of the left eye, the late angiography (**A**) shows optic disc hyperfluorescence and pooling. Infrared imaging (**B**) and fundus photography (**C**) show the Paton’s folds (yellow arrow). B-scan through the fovea in the acute phase (**D**). In the non-acute phase, the *en face* OCT shows the "fingerprint sign" and corresponding B-scan at the level of OPL/HFL reveal a *dentate* or *saw-tooth* appearance (**E**)

**Fig. 4:**
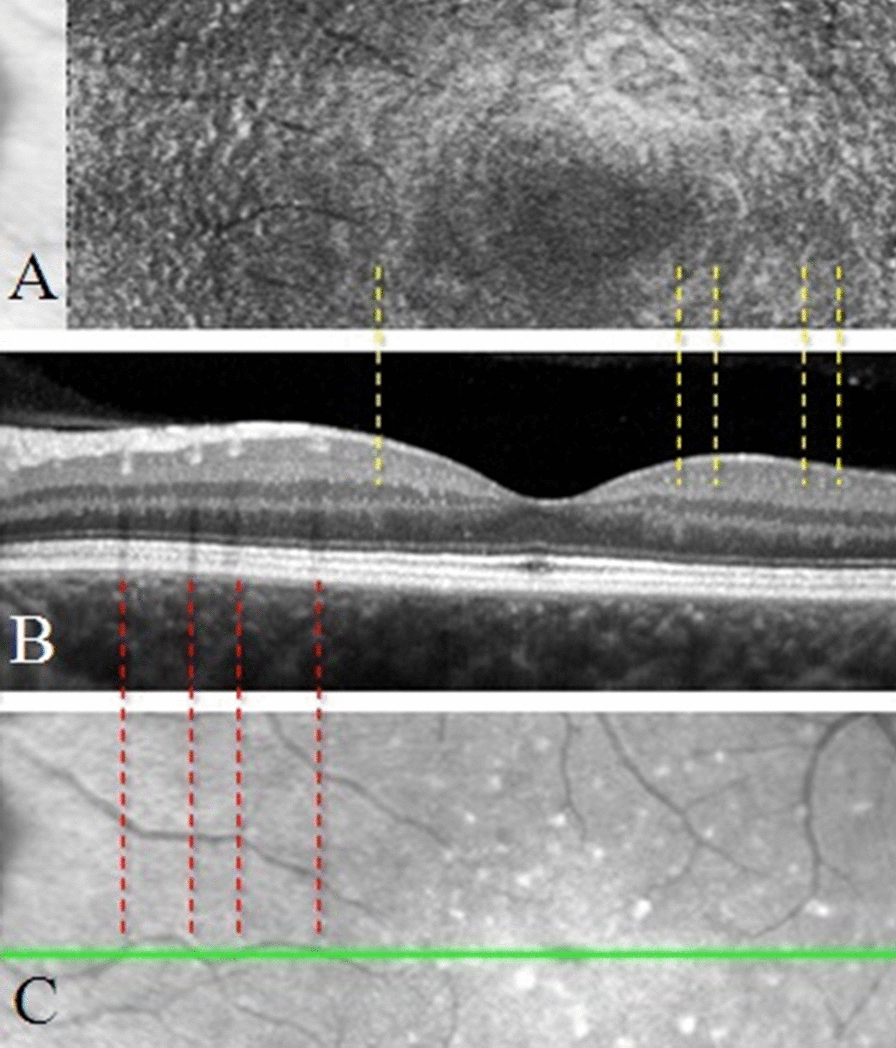
(Case 3): The images **A**, **B** show that the *dentate* appearance corresponds to the hyper reflective concentric lines in the "fingerprint sign" (yellow dotted lines). Retinal vessels (red dotted lines) are used as reference for *en face* OCT image and of the B-scan alignment (**C**)

A 42-year-old woman presented with 21 days of bilateral scotomas associated with blurred vision, frontal headache, and tinnitus. The time between the onset of symptoms and the start of treatment was 23 days. On presentation, Paton's folds were visualized in OS. Visual acuity at the initial assessment was counting fingers OU and 20/20 OU after one month of corticosteroid therapy. She was maintained on low-dose corticosteroid monotherapy for 24 months. The *dentate* appearance OU at the OPL/HFL interface was first seen in the first month OU. Concentric rings or waves at the level of the OPL/HFL interface were first seen when OCT reconstruction was performed following 5 years of disease quiescence (Figs. 3 and 4).

## Discussion

The use of OCT and the advancement of this technology has allowed clinicians to observe new changes in the retinal architecture in VKHD [5, 7]. Analyzing the volumetric images of the *en face* OCT reconstructions at the level of the OPL/HFL with a *dentate* or *saw-tooth* pattern in the OCT B-scan, we observed findings similar in appearance to the fingerprint described in other non-inflammatory conditions [8, 9]. In agreement with the literature, we believe that the images of the concentric waves seen in the volumetric OCT slab resemble the appearance of ridges and valleys, which are the result of disruption of the normally highly aligned photoreceptor axons and associated Müller cells, as proposed in eyes with vitreomacular traction disorders [8].

Diverse mechanisms appear to result in a shared OCT signal, reflecting the innate distensibility of HFL and associated OPL. Not only does the anterior traction caused by the epiretinal membrane induce the formation of the wavy aspect in OPL/HFL interface, but lateral vector forces appear to be capable of producing a similar finding [8, 10]. In accordance with this HFL distensibility hypothesis, Wolter proposed a mechanism for OPL/HFL waviness, histologically identified in a case of orbital tumor, in which postero-anterior compressive forces were transmitted to the retina, forming opposite folds of the internal choroid and RPE of the foveal region. The author believes that the HFL may have acted as a “breakwater” between the choroid's horizontal folds and the retina's foveal vertical folds, reshaping these coarse wrinkles in opposite directions into small and orderly waves that acted to maintain the foveola's normal shape and order [11]. Patients with macular edema have shown similar histological findings. This regular, *dentate* reorganization of the OPL/HFL might be a mechanism to allow for more even distribution of fluids within the tissues, thereby preventing force vectors on the vertical axis from pulling on the foveolar cones and preserving central vision [12].

In the acute phase of VKHD, choroidal thickening, as well as the accumulation of exudative subretinal fluid and, less commonly, the presence of macular edema, can cause a postero-anterior distortion, resulting in disruption of the OPL/HFL. These changes at the level of the OPL/HFL interface may persist even following resolution of the acute phase as observed in our cases. Bulging of the choroid, described in our previous report, could also contribute to distortion of the OPL/HFL, but no choroidal bulging was observed in any patient of this series [13]. The breakdown of the blood-retinal barrier in the inflammatory process causes vascular leakage, allowing the serum to infiltrate into the retinal interstitium [14]. VKH disease is a primary autoimmune stromal choroiditis and the eyes with SRD, fluorescein angiography shows multiple hyperfluorescent points in the early phases due to breaks in the pigment epithelium barrier. Some authors also describe perivascular leakage as a finding of the disease [15, 16]. The disturbance of the RPE barrier and accumulation of subretinal exudative fluid in the acute phase of the disease could be an explanation for the involvement of OPL/HFL by the intraretinal edema.

Lateral or tangentially oriented distortion causing the “fingerprint sign” on *en face* OCT is exemplified by Paton's folds seen as peripapillary curvilinear lines in diseases with optic disc edema. The use of OCT has allowed the characterization of 3 different peripapillary folds: superficial wrinkles at the level of retinal nerve fiber layer, outer retinal folds involving all the layers from outer plexiform layer to ellipsoid zone, and choroidal folds. These folds are formed as a result of the lateral compression mechanism caused by the bulging of the optic disc, as well as by the complex interaction between the properties of the tissue and force vectors. Outer retinal folds in the peripapillary region are frequently associated with subretinal fluid. As the papilledema resolves, they may form creases that correspond to "high water marks" seen in the ophthalmoscopy. They can also extend further into the peripapillary area, reaching the macula [10].

Ramtohul et al. described concentric macular rings in subjects with foveal hypoplasia. In addition to the *dentate* appearance in the B-scan and the undulations in the *en face* OCT at the level of OPL/HFL*,* the authors observed the concentric rings on color fundus photography, which were not found in our series of cases. A different proposal from that presented in the literature for the “fingerprint sign”, disorders of foveal maturation, results in a greater number of vertically oriented HFL and the lack of a radial pattern in the center, explaining the different geometry of the OPL/HFL interface in these subjects [9].

It is of note that the angle of the measurement beam, off-axis, can impact the visualization of the OPL/HFL in the B-scan as in Fig. 1. This characteristic is due to the perpendicularity of the measuring beam in this layer [17, 18]. Interestingly, the visualization of the *dentate* or *saw-tooth* pattern was not affected, and the finding was visualized on the tilted up and tilted down sides of the cross-sectional macular images.

Among the limitations of this study are the retrospective analysis of cases with the potential for selection bias, the small sample size, and the fact that *en face* OCT reconstruction was added to the protocol in 2019, which prevented us from determining the exact timing of when the “fingerprint sign” appeared.

## Conclusions

To our knowledge, this is the first description of an inflammatory disease causing such a *dentate* appearance on cross-sectional B-scan and the undulations in the *en face* OCT reconstruction at the level of the OPL/HFL. Among previously proposed pathogenic mechanisms, distortion caused by choroidal thickening, SRD, and/or retinal edema would seem to be the most likely, which, even following resolution, leaves residual disruption of the OPL/HFL. Future studies with longitudinal data, a larger sample size, evaluation in other inflammatory diseases, and additional functional and anatomical evaluations may help to understand the nature and prognostic value of these findings.

## Data Availability

The datasets used and analysed during the current study are available from the corresponding author on reasonable request.

## Abbreviations

CNV: Choroidal neovascular membrane
EDI OCT: Enhanced depth imaging optical coherence tomography
HCFMUSP: Hospital das Clinicas da Faculdade de Medicina da Universidade de Sao Paulo
HFL: Henle fiber layer
OCT: Optical coherence tomography
OCTA: Optical coherence tomography angiography
OD: *Oculus dexter*
OPL: Outer plexiform layer
OS: *Oculus sinister*
OU: *Oculus uterque*
RPE: Retinal pigment epithelium
SD OCT: Spectral-domain optical coherence tomography
SRD: Serous retinal detachments
VKHD: Vogt-Koyanagi-Harada disease
